# Molecular Studies of HTLV-1 Replication: An Update

**DOI:** 10.3390/v8020031

**Published:** 2016-01-27

**Authors:** Jessica L. Martin, José O. Maldonado, Joachim D. Mueller, Wei Zhang, Louis M. Mansky

**Affiliations:** 1 Institute for Molecular Virology, Pharmacoimmunology Training Program & Pharmacology Graduate Program, University of Minnesota, 18-242 Moos Tower, 515 Delaware Street SE, Minneapolis, MN 55455, USA; mart3243@umn.edu; 2 Institute for Molecular Virology & DDS-PhD Dual Degree Program, University of Minnesota, 18-242 Moos Tower, 515 Delaware Street SE, Minneapolis, MN 55455, USA; jmaldo@umn.edu; 3 Institute for Molecular Virology & School of Physics and Astronomy, University of Minnesota, 18-242 Moos Tower, 515 Delaware Street SE, Minneapolis, MN 55455, USA; mueller@physics.umn.edu; 4 Institute for Molecular Virology, School of Dentistry & Characterization Facility, University of Minnesota, 18-242 Moos Tower, 515 Delaware Street SE, Minneapolis, MN 55455, USA; zhangwei@umn.edu; 5 Institute for Molecular Virology, School of Dentistry & Pharmacology Graduate Program, University of Minnesota, 18-242 Moos Tower, 515 Delaware Street SE, Minneapolis, MN 55455, USA

**Keywords:** deltaretrovirus, antiretroviral, lentivirus

## Abstract

Human T-cell leukemia virus type 1 (HTLV-1) was the first human retrovirus discovered. Studies on HTLV-1 have been instrumental for our understanding of the molecular pathology of virus-induced cancers. HTLV-1 is the etiological agent of an adult T-cell leukemia (ATL) and can lead to a variety of neurological pathologies, including HTLV-1-associated-myelopathy/tropical spastic paraparesis (HAM/TSP). The ability to treat the aggressive ATL subtypes remains inadequate. HTLV-1 replicates by (1) an infectious cycle involving virus budding and infection of new permissive target cells and (2) mitotic division of cells harboring an integrated provirus. Virus replication initiates host antiviral immunity and the checkpoint control of cell proliferation, but HTLV-1 has evolved elegant strategies to counteract these host defense mechanisms to allow for virus persistence. The study of the molecular biology of HTLV-1 replication has provided crucial information for understanding HTLV-1 replication as well as aspects of viral replication that are shared between HTLV-1 and human immunodeficiency virus type 1 (HIV-1). Here in this review, we discuss the various stages of the virus replication cycle—both foundational knowledge as well as current updates of ongoing research that is important for understanding HTLV-1 molecular pathogenesis as well as in developing novel therapeutic strategies.

## 1. Introduction

Human T-cell leukemia virus type 1 (HTLV-1) was independently discovered in 1980 by two research groups and identified as the etiological agent of an adult T-cell leukemia (ATL) [1,2]. As the first human retrovirus discovered, research on HTLV-1 laid the foundational framework for subsequent studies of human immunodeficiency virus type 1 (HIV-1), infectious causes of cancer, and the molecular mechanisms of leukemogenesis [3].

Shortly after the discovery of HTLV-1, another human retrovirus was discovered—human T-cell leukemia virus type 2, HTLV-2—which closely resembled HTLV-1 in genome structure and nucleotide sequence [4]. Unlike HTLV-1, HTLV-2 has not been convincingly associated with human pathology. Nevertheless, both HTLV-1 and HTLV-2 are included in worldwide prevalence estimates. Historically, it has been estimated that 15–20 million people are infected worldwide [5,6]. A more recent study has estimated the number closer to 5–10 million, with the majority of these individuals residing in Japan and the Caribbean Basin [7]. A third and fourth type of HTLV, human T-cell leukemia virus type 3 (HTLV-3) and human T-cell leukemia virus type 4 (HTLV-4), have been discovered in central Africa in the past decade; both are closely related to HTLV-1, and likely share similarities in replication, pathogenesis and transmission [8,9]. 

HTLV-1 is the etiological agent of ATL as well as a variety of neurological pathologies, primarily HTLV-1-associated-myelopathy/tropical spastic paraparesis (HAM/TSP) [10]. Both ATL and HAM/TSP have a low incidence among HTLV-1 carriers. It is thought that approximately 2%–6% of patients infected with HTLV-1 will acquire either pathology [11,12]. ATL generally presents after a long latency in patients infected during childhood. This is in contrast to HAM/TSP, which is associated with infection later in life [13].

ATL is an aggressive malignancy of the peripheral T-cells and can be divided into four subtypes—acute, lymphomatous, chronic, or smoldering. Patients with the acute form of ATL have a prognosis of approximately 6 months—an estimate that has not significantly changed since the discovery of the disease, despite advances in treatments [14]. Current recommended therapies for ATL include chemotherapy, monoclonal antibodies, allogeneic bone marrow transplants, and a combination of interferon-α (IFN-α) and azidothymidine (AZT) [15,16,17,18]. Interestingly, the mechanism of action of the combination of IFN-α and AZT appears to correlate with an induction of cell apoptosis by phosphorylation of p53 [19].

HAM/TSP is characterized by spasticity and weakness of the legs along with urinary disturbances [19]. The primary pathology of HAM/TSP is associated with HTLV-1 infection in the spinal cord leading to inflammation. Unlike ATL, which appears to have a complex and multi-faceted pathology, the incidence of HAM/TSP has been shown to correlate with HTLV-1 proviral loads as well as the site of proviral integration [20,21]. Treatment of HAM/TSP is symptom-based and includes antispasmodic and anti-inflammatory medications [22].

Research into the HTLV-1 life cycle to date has been essential in the discovery and development of better therapeutic strategies. Here in this review, we highlight what is currently known as well as recent advances in the study of HTLV-1 replication. The recent advances help to provide further reason for hope in effective therapeutic options for HTLV-1-infected individuals.

## 2. HTLV-1 Infectious Replication Cycle

### 2.1. Attachment and Fusion

**Figure 1:**
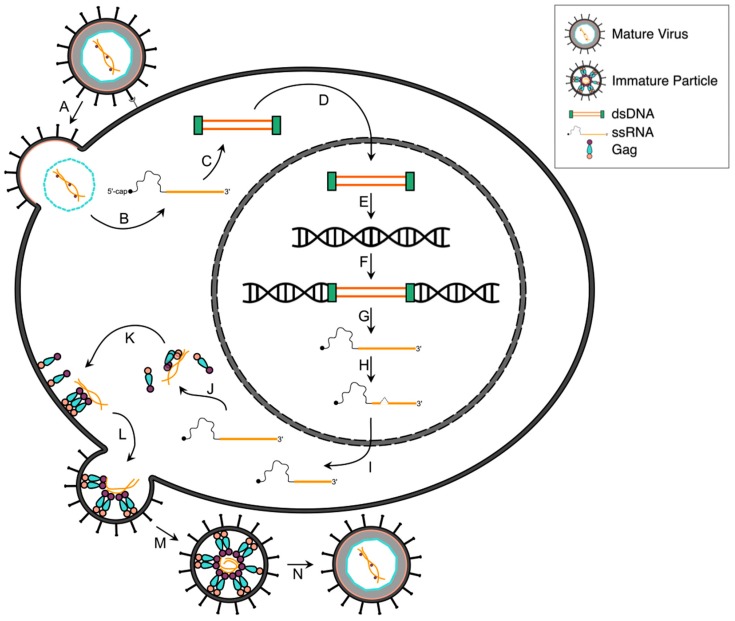
HTLV-1 life cycle. The major steps in the life cycle of HTLV-1 are shown. A mature, infectious HTLV-1 virion attaches and fuses to the target cell membrane through interaction with the target cell surface receptors GLUT1/HSPG/NRP-1 via the HTLV-1 envelope surface and transmembrane domains of the envelope (Env) protein (**A**). Following fusion, the viral core containing the viral genomic RNA (gRNA) is delivered into the cytoplasm (**B**), and during and/or following entry the gRNA genome undergoes reverse transcription to convert the gRNA into double stranded DNA (dsDNA) (**C**). The dsDNA is then transported into the nucleus (**D**), and it is integrated into the host genome; (**E,F**). The provirus is then transcribed by cellular RNA polymerase II (**G**), as well as post-transcriptionally modified (**H**). Both full-length and spliced viral mRNAs are exported from the nucleus to the cytoplasm (**I**). The viral proteins are then translated by the host cell translation machinery (**J**), and the Gag, Gag-Pol and Env proteins transported to the plasma membrane (PM) along with two copies of the gRNA genome (**K**). These viral proteins and gRNA assemble at a virus budding site along the PM to form an immature virus particle (**L**). The budding particle releases from the cell surface (**M**), and undergoes a maturation process through the action of the viral protease, which cleaves the viral polyproteins to form an infectious, mature virus particle (**N**).

HTLV-1 primarily infects CD4^+^ T-cells but has the potential to infect a wide variety of cells, including CD8^+^ T-cells, B-lymphocytes, endothelial cells, myeloid cells, fibroblasts, as well as other mammalian cells [23,24,25,26,27]. This wide variety of target cells is due in part to the ability of the surface subunit (SU) of the HTLV-1 envelope glycoprotein (Env) to interact with three widely distributed cellular surface receptors including the glucose transporter (GLUT1) [28], heparin sulfate proteoglycan (HSPG) [29], and the VEGF-165 receptor neuropilin-1 (NRP-1) [30]. Once HTLV-1 has attached to the cell, the membrane fusion process occurs by a series of proposed sequential events between SU and the target cell receptor proteins (Figure 1A,B) [30,31]. Briefly, the HTLV-1 Env interacts with HSPG first followed by NRP-1, which results in the formation of a complex. Following this event, GLUT1 associates with the HSPG/NRP-1 complex to initiate the fusion process, through interactions with the HTLV-1 Env transmembrane (TM) protein, which allows for the HTLV-1 capsid (CA) core containing the viral genome and viral proteins to be released into the cytoplasm of the permissive target cell (Figure 1B).

It has been demonstrated that GLUT1 plays a key role in both the binding of the SU and the infection of CD4^+^ cells [32]. Paradoxically, other retroviruses have mechanisms that decrease surface expression of their receptors, such as HIV-1 Nef and Vpu [33,34]. The decrease of receptor expression on the cell surface is thought to prevent both superinfection and intracellular Env-receptor interactions, which can inhibit proper proteolytic processing of the Env precursor polyprotein. HTLV-1 does not encode for an accessory protein that reduces surface expression of GLUT1, and it is therefore unclear how HTLV-1 modulates plasma membrane receptor expression. However, it has been recently shown that HTLV-1-based virus-like particles (VLPs) produced in cells with high levels of GLUT1 were better able to fuse with target cells than those produced from cells with low levels of GLUT1 [35]. In 293T cells, HTLV-1 Env avoids interaction with GLUT1 through the separate intracellular localization of GLUT1 and Env [35]. This recent observation is important because it suggests that separate intracellular localization of GLUT1 and HTLV-1 Env is required for proper fusion activity of the HTLV-1 Env. This study may also have implications for HTLV-1 cellular tropism, as CD4^+^ regulatory T-cells, the primary viral reservoir for HTLV-1-infected individuals, express GLUT1 at low levels as compared to other types of CD4^+^ T-cells [36].

### 2.2. Reverse Transcription, Nuclear Transport and Integration

The HTLV-1 CA core enters the infected cell and contains two copies of the viral genomic RNA (gRNA) along with reverse transcriptase (RT), integrase (IN), and the viral protease (PR). Reverse transcription of HTLV-1 RNA to double-stranded DNA (dsDNA) has not been extensively studied but likely occurs after virus entry (Figure 1C) [37,38]. It is thought that HIV-1 reverse transcription is linked to intracellular uncoating of the CA core [39]. Additionally, HIV-1 RT and IN interactions have been shown to be necessary for production of early reverse transcription products [40]. Complementary studies with HTLV-1 have yet to be done, so it is unclear whether HTLV-1 CA uncoating correlates with reverse transcription or if RT-IN interactions occur during early reverse transcription. Recombination can occur during reverse transcription, and recent evidence from phylogenetic analyses strongly suggests that recombination played a distinct role in emergence of HTLV-1 in the human population approximately 4000 years ago [41]. 

Unlike HIV-1, which is highly sensitive to the effects of the APOBEC family of cytidine deaminases, HTLV-1 appears less sensitive APOBECs. There is some evidence that APOBEC3G may lead to G-to-A hypermutation in some HTLV-1 sequences *in vivo* [42,43], but the overall effect on HTLV-1 sequence diversity appears to be negligible—perhaps due to the propensity of HTLV-1 to be propagated by clonal expansion of infected cells rather than replication via reverse transcription. HTLV-1 has been previously shown to prevent APOBEC3G packaging through an element at the C-terminal nucleocapsid (NC) region of Gag [44].

The partially disassembled core containing the reverse transcription complex (preintegration complex) is translocated to the nucleus (Figure 1D) where integration into the host cell chromosome occurs to form the provirus (Figure 1E,F). It has been found that HTLV-1 integrates into the genome in the absence of preferred sites [45,46,47,48,49,50]. Such studies have analyzed hundreds of thousands of HTLV-1 integration sites [51,52] and have not been able to identify HTLV-1 proviral integration site hotspots. Interestingly, in HTLV-1-induced disease states, the integration sites of HTLV-1 become non-random. For example, it was recently demonstrated that the clinical diagnosis of HAM/TSP correlates with proviral integration into transcriptionally active regions [53].

### 2.3. Viral Gene Transcription

The long terminal repeats (LTRs) of the HTLV-1 provirus contain the necessary promoter and enhancer elements to initiate RNA transcription (Figure 1G), with the polyadenylation signal located in the 3′LTR [1]. Tax, a non-structural protein and the main driver of viral transcription, potently activates viral transcription during the early phase of infection by recruiting multiple cellular transcription factors [54]. Three conserved 21-bp repeat elements, known as the Tax-responsive element 1 (TRE-1), bind the cyclic AMP response element binding protein (CREB) at the TRE-1 site through its N-terminus (NTD) [55,56,57,58,59,60,61], while the C-terminal domain (CTD) of Tax is believed to promote the transcriptional initiation and RNA polymerase elongation by directly interacting with the TATA binding protein [5,62]. The Tax-CREB promoter complex recruits the multifunctional cellular coactivators CREB binding protein (CBP), p300, and the p300/CBP-associated factor to the LTR [63,64,65,66,67,68].

Recently, several host factors that directly interfere with HTLV-1 viral transcription have been identified. TCF1 and LEF1 are transcription factors specifically found in T-cells. They antagonize Tax activity through physical association with Tax, preventing transcription of the viral proteins. In most HTLV-1-infected cell lines, however, TCF1 and LEF1 expression is low due to downregulation via STAT5a, which is activated by Tax [69]. The host protein SIRT1 deacetylase has also been shown to downregulate HTLV-1 viral transcription by inhibiting Tax. Unlike TCF1 and LEF1, SIRT1 appears to inhibit Tax-CREB interactions. Interestingly, the well-known SIRT1 activator resveratrol significantly decreases the transmission of HTLV-1 produced from MT2 cells [70,71]. This suggests that resveratrol may be a potential therapeutic option for patients infected with HTLV-1 or a prophylactic option to prevent virus transmission. In addition to these cellular host factors, the facilitate chromatin transcription (FACT)proteins SUPT16H and SSRP1 have been shown to inhibit both HTLV-1 and HIV-1 transcription by preventing interaction of HTLV-1 Tax and HIV-1 Tat with their respective viral LTRs [72].

### 2.4. Post-Transcriptional Regulation

Rex is a positive post-transcriptional regulator essential for splicing and transport of HTLV-1 mRNA (Figure 1H,I). Rex specifically interacts with the U3 and R regions of the HTLV-1 gRNA known as the Rex-responsive element (RexRE). During the early stages of viral gene transcription, suboptimal levels of Rex are present [73], which results in the exclusive export of doubly spliced (*tax, rex*, *p30II*, *p12*, *p13*, and *hbz*) viral mRNAs to the cytoplasm (Figure 1I) [74]. Once Rex accumulates in the nucleus, Rex reduces splicing of viral mRNA and the singly spliced (*env*) and unspliced (*gag-pro-pol*) mRNAs are then exported from the nucleus to the cytoplasm leading to the production of enzymatic and structural proteins (Figure 1J) [74]. Rex binds to the RexRE through a highly basic RNA-binding NTD, while the CTD is important for protein oligomerization [75,76]. Rex also contains an activation domain containing the nuclear export signal, which targets Rex to the nuclear pore complex in order for Rex to move between the nucleus and cytoplasm [77,78].

Despite the presence of host cell mechanisms to export doubly spliced RNA, all HTLV-1 mRNA transcripts, including those that are doubly spliced, have RexREs present. A recent study has shown that Rex may have a CRM1-dependent role in nuclear export of all HTLV-1 mRNAs, even the doubly spliced mRNAs that should be exported via host cell mechanisms [79]. These observations suggest that viral mRNA export is under a more complex regulation than previously thought. Furthermore, another recent study has suggested that there are three alternatively spliced HTLV-1 transcripts that encode for novel Rex isoforms, which may also contribute to the regulation of HTLV-1 protein expression levels [80].

### 2.5. Viral Protein Translation

As soon as HTLV-1 mRNAs are exported to the cytoplasm, the host protein-synthesis machinery translates the viral proteins. Presumably, the full-length viral gRNA is either translated or trafficked to the plasma membrane, where it can dimerize, interact with the Gag polyprotein, and be packaged into assembling particles (Figure 1K,L) [81]. The doubly spliced and unspliced mRNAs are translated by free ribosomes to express the enzymatic and structural proteins, respectively, while the singly spliced mRNA is translated by membrane-bound ribosomes to express Env [45].

Many RNA viruses use a cap-independent mechanism to recruit the 40S ribosomal subunit to an internal ribosome entry segment (IRES) within the 5′UTR of the mRNA, which allows for ribosomal scanning and protein translation to occur [82,83,84]. It was thought that HTLV-1 mRNA contains an IRES element [85] used for the translation of the Gag protein, but another study suggests that a 5′ proximal post-transcriptional control element modulates post-transcriptional HTLV-1 gene expression by interacting with the host RNA helicase A instead of an IRES element, implying that the translation of the HTLV-1 mRNA is cap-dependent [86]. Interestingly, a recent study has demonstrated that HTLV-1 translation is inhibited by the drug edeine, a cap-independent translation inhibitor, suggesting that an IRES element in the 5′ UTR recruits the ribosome to the mRNA [87]. Obviously, more research is needed to firmly establish the mechanism(s) used by HTLV-1 to translate its viral proteins.

### 2.6. Gag and Viral RNA Trafficking

Viral particle formation occurs after Gag traffics from the cytoplasm to the plasma membrane (PM) (Figure 1K). How HTLV-1 Gag translocates from the site of translation to the membrane is poorly understood. However, it is known that monomeric forms of HTLV-1 Gag exist in the cytoplasm and are detected at the membrane shortly after the initiation of viral protein translation [88]. This is in contrast to HIV-1 Gag, where low ordered oligomers are observed in the cytoplasm until micromolar concentrations are reached prior to detecting oligomeric Gag at the plasma membrane [88]. HIV-1 Gag interacts with many cellular proteins, including cytoskeleton-associated proteins, though their relationship to HIV-1 Gag trafficking is unclear [89]. HTLV-1-infected cells regulate cytoskeletal polarization [90], though it is unclear if this is related to Gag trafficking to the plasma membrane. 

HTLV-1 Gag nucleocapsid (NC) protein binds to HTLV-1 RNA relatively weakly as compared to that of other retroviral NC proteins, due in part to the anionic carboxy-terminal domain (CTD) of the HTLV-1 NC [91]. The HTLV-1 MA has been recently reported to bind RNA, and it was found that HTLV-2 MA binds RNA at much higher affinity than HTLV-2 NC [92]. This is in direct contrast to HIV-1, in which NC binds to RNA more strongly than HIV-1 MA [92]. These recent findings highlight the importance of both the MA and NC domains in viral RNA interactions that are likely critically important for viral gRNA recognition and gRNA packaging. How HTLV-1 RNA traffics through the cytoplasm in order to get to the plasma membrane (and to virus budding sites) is poorly understood, but a recent study with HIV-1 gRNA suggests that the viral gRNA diffuses through the cytoplasm to the membrane [93]. It is formally possible that HTLV-1 gRNA also diffuses through the cytoplasm to reach the membrane, but it could also bind to Gag before reaching the membrane (Figure 1I–K). There is a significant need for future studies in order to better understand these aspects of HTLV-1 replication.

### 2.7. Assembly, Budding and Maturation

Gag-gRNA, Gag-Gag and Gag-membrane interactions are all required for the assembly and budding of virus particles (Figure 1L) [94]. Gag forms higher order oligomers by oligomerizing with other Gag molecules through interactions primarily involving the CA domain and to some extent the NC domain [95,96,97,98,99,100]. Once at the PM, virus budding sites are identified and are characterized by the interaction of HIV-1 MA with lipid-rich [101] assembly sites known as lipid rafts [102,103,104]. Membrane binding of HIV-1 Gag is dependent upon interaction of MA with phosphatidylinositol-(4,5)-bisphosphate PI(4,5)P_2_ [105]. HTLV-1 Gag has been shown to not have a preference for binding to PI(4,5)P_2_, which has implications for how HTLV-1 Gag targets the PM and identifies virus budding sites [105]. Cellular factors are also recruited to the virus budding sites, resulting in budding and subsequent release of immature virus particles (Figure 1L,M) [100,106,107]. The viral protease (PR) cleaves the Gag and Pol polyproteins during and shortly after the release of immature virus particles (Figure 1N) [108]. MA remains closely associated with the PM; CA forms a capsid shell that contains reverse transcriptase, integrase and the NC-coated gRNA. The mature virus particle, if infectious, is capable of infecting a permissive target cell (Figure 1N) [109].

## 3. HTLV-1 Transmission

### 3.1. Inter-Host Transmission

There are generally three modes of inter-host HTLV-1 transmission described: (1) blood and blood products, (2) vertical or (3) sexual transmission [110], but the main mode of transmission is thought to be vertical, *i.e.* from mother-to-child through breastfeeding [111]. Mother-to-child transmission rates vary from 5% to 27% for children nursed by infected mothers and correlate with the duration of breastfeeding [112,113]. While it is not clear precisely how infection occurs through the mucosal and epithelial barriers of the gastrointestinal tract, it is thought that infected lymphocytes in breast milk carry the virus into the gut [114]. Once in the gut, either cell-free virus or cells carrying the virus must pass through the epithelium. A recent study demonstrated *in vitro* that cell-free HTLV-1 may cross the epithelial barrier via transcytosis before infecting subepithelial dendritic cells [115]. The precise mechanism of transcytosis for HTLV-1 remains unclear. However, studies with HIV-1 have shown that transcytosis across vaginal epithelial cells occurs via the endocytic recycling pathway [116]. It is plausible that other mechanism(s) are involved in HTLV-1 infection across the gut epithelial barrier due to the low infectivity of cell-free virus. While cell-free HIV-1 is generally thought to be much more infectious than cell-free HTLV-1, it has been suggested that HIV-1-infected lymphocytes more efficiently infect target cells in the gut than cell-free virus – possibly through the formation of a viral synapse that induces transcytosis [117]. The role of the virological synapse in these transmission events has not been carefully studied. It is also not known whether HTLV-1 infected lymphocytes can transmigrate as a whole cell across the epithelial barrier and infect subepithelial immune cells.

Zoonotic transmission events of simian T-cell leukemia virus type 1 (STLV-1) to humans after contact with nonhuman primates through bites or bushmeat slaughtering still occur in Africa, establishing the emergence of new HTLV-1 infections in humans. A recent study found that more than 8% of individuals bitten by nonhuman primates in Africa are infected with HTLV-1, and virus transmission cannot be attributed to mother-to-child transmission [118]. The strains of HTLV-1 found in those infected closely resembled the subtypes of STLV-1 commonly found in the primate species from which they were bitten [118,119]. In fact, it is likely that the emergence of HTLV-3 and HTLV-4 may be attributable to recent STLV zoonotic transmission events, as STLV-4 is known to be endemic in African gorillas, and phylogenetic analyses have shown that HTLV-4 is not an ancient human virus but recently emerged in the human population [120]. While these findings highlight the potential ongoing role of nonhuman primates as virus reservoirs, they also highlight interest in the virus-host interactions that facilitate cross-species transmission as well as potential risks in transmission and emergence of more highly pathogenic types of HTLV. While monkeys in Japan also harbor STLV-1 strains [121], those strains are more highly divergent from the HTLV-1 strains in Japanese patients, indicating that zoonotic transmission of HTLV-1 may not be a major public health threat in regions outside of Africa [122].

### 3.2. Cell-to-Cell Transmission

In general, there are two distinct methods of virus transmission between cells: virus infection of cells in the absence of cell-to-cell contacts and virus infection involving cell-to-cell contacts. Most retroviruses can efficiently infect target cells in the absence of cell-to-cell contacts—in which the virus buds from the cell and infects a target cell through diffusion. HTLV-1 is notorious for being poorly infectious in the absence of direct cell-to-cell transmission, and co-cultivation of permissive target cells with virus-producing cells are the most effective means of virus transmission [123].

### 3.3. Virological Synapses

**Figure 2:**
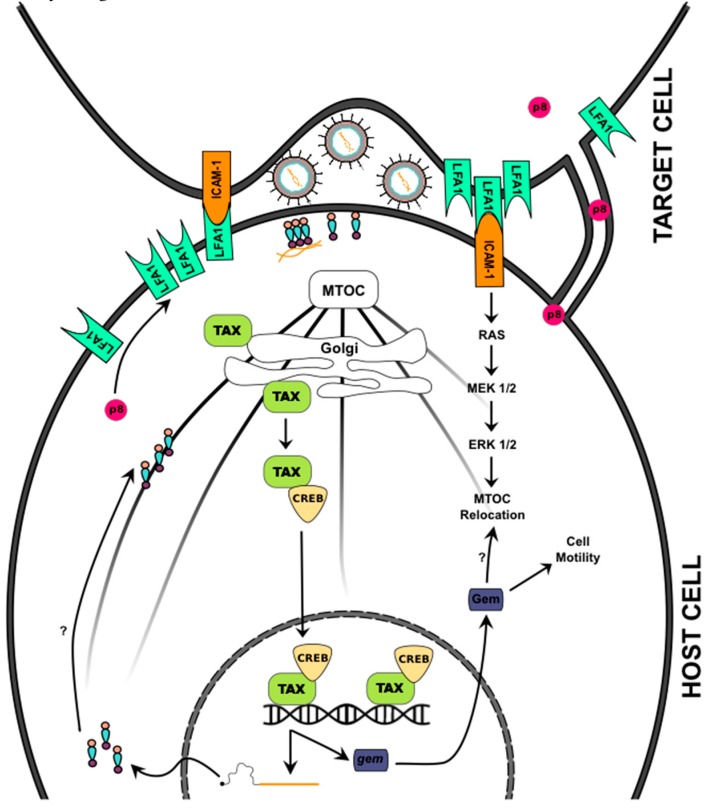
HTLV-1 Cell-to-Cell Transmission. Shown in the diagram is a host cell (**bottom**) that has anchored itself to a permissive target cell (**top**) using ICAM-1 and LFA1. The HTLV-1 accessory protein p8 has been shown to increase the expression of the LFA1 receptors as well as increase the number of cell-cell synapses, which p8 then traffics through to increase LFA1 in the target cell. Once ICAM-1 is bound, it triggers a signaling cascade that leads to the relocation of the MTOC. Additionally, HTLV-1 Tax is found bound to Golgi bodies that are attached to the MTOC. This initiates a variety of cell signaling cascades. For example, one established pathway increases expression of Gem, a protein that increases cell motility and possibly MTOC relocation. As HTLV-1 Gag is produced, it is found concentrated at the MTOC. Subsequently, HTLV-1 Gag is found at the sites of cell-cell contacts where the virus particles bud into the viral synapse formed by the cellular adhesion points. Virus particles produced can bind receptors for entry into the permissive target cell. The question marks along the lines with arrows indicate mechanisms of transport or activation that are not well understood.

Immunofluorescence and confocal microscopy were used previously to demonstrate that Gag and Env proteins are more evenly distributed in isolated T-cells, but once the cell comes into contact with another cell, cell polarization occurs—impacting the localization of HTLV-1 Gag, Env and the genomic RNA towards the cell-cell junction. This cell-to-cell junction, termed the virological synapse (VS), shares many features with the previously described immunological synapse, which includes features such as ordered talin domains and microtubule organizing center (MTOC) polarization [124]. Cryoelectron tomography studies of HTLV-1 associated VS structures suggest that there is no fusion of the cell membranes [125]. To the contrary, HTLV-1 transmission occurs via rapid budding and fusion of the HTLV-1 virus across the VS from the infected to uninfected cell (Figure 2).

It has been reported that the formation of the VS is triggered by HTLV-1 infection and is not dependent on signaling through the T-cell receptor as is seen in immunological synapses [124]. The VS forms when the surface adhesion molecule intercellular adhesion molecule-1 (ICAM-1) is engaged by its ligand lymphocyte function-associated antigen 1 (LFA-1) [90,126]. ICAM-1 then activates the MEK/ERK pathway, which contributes to MTOC relocation. The HTLV-1 Tax protein, the key virus transcription accessory protein, works in synergy with ICAM-1 to facilitate MTOC polarization. While it is primarily a nuclear protein, Tax can be found in the cytoplasm near the MTOC as well as in the cell-cell contact region [127]. Tax activates the CREB-signaling pathway during the formation of the HTLV-1 VS [126]. The CREB pathway increases expression of Gem, a small GTP-binding protein in the RAS superfamily, which is involved in cytoskeleton remodeling and cell migration [128]. Tax also appears to upregulate ICAM-1 in HTLV-1-infected cells, indicating that ICAM-1 and Tax appear to have synergistic roles in HTLV-1 VS formation [129].

When comparing HTLV-1 cell-cell transmission to HIV-1 cell-cell transmission, HTLV-1 is more dependent on cell contact for infectivity. Coculturing of virus-producing cells with permissive target cells significantly enhances HTLV-1 transmission by several thousand-fold, but only increases HIV-1 transmission 10–100 hundred-fold [130,131]. Furthermore, disruption of actin and tubulin polymerization inhibits HTLV-1 spread to a greater extent than that of HIV-1 spread [131]. Nonetheless, HIV-1 cell-cell transmission remains a highly relevant form of virus spread *in vivo*. A recent study helps to highlight this through the observation that granulocytes (e.g., basophils) may actually capture HIV-1 and contribute to virus transmission [132].

### 3.4. Viral Biofilms

In addition to transmission via the VS, HTLV-1 particles have been reported to have the ability to form a biofilm-like, carbohydrate-rich extracellular structure on the surface of cells. These structures are composed of collagen, agrin, tetherin, and galectin-3 and may function as a way to concentrate HTLV-1 particles in a single location to increase the likelihood of infection of a permissive target cell [133]. A recent report described the creation of antiviral biofilm monoclonal antibodies using purified biofilms from MT2 cells. It was found that in addition to the structural proteins previously identified, the antigens CD4, CD150, CD25, CD70, and CD80 were also identified in these viral biofilms. Tax expression was found to modulate the level of these antigens in the viral biofilm [134].

While it has been observed that dendritic cells may be infected by cell-free HTLV-1 *in vitro* [31], a recent study analyzed the infectivity of chronically infected C91PL cell culture supernatant as compared to purified biofilms in primary human monocyte-derived dendritic cells (MDDCs) and T lymphocytes. It was found that while MDDCs were more easily infected than were T lymphocytes, both cell types achieved significantly higher proviral loads when exposed to viral biofilms as opposed to supernatant-derived virus [135].

## 4. Monoclonal Expansion of HTLV-1 Infected Cells and Leukemogenesis

While HTLV-1 cell-cell transmission is likely a critical determinant of virus transmission from an infected individual to a susceptible individual, many studies have established that the primary route of replication for HTLV-1 *in vivo* is through mitotic division of host cells and subsequent propagation of the provirus by clonal expansion. DNA analysis of HTLV-1 sequences from patients in geographically distinct locations shows very little genetic variation among HTLV-1 isolates [136]. This is likely because of a low evolutionary rate of 7.06 × 10^−7^–1.38 × 10^−5^ substitutions per site per year in the *LTR* and *env* regions [137]. While the HTLV-1 reverse transcriptase has a reduced mutation rate as compared to HIV-1 reverse transcriptase (7 × 10^−6^ mutations per target base pair per replication cycle for HTLV-1 as compared to 3.4 × 10^−5^ for HIV-1), the fourfold difference is likely not sufficient alone to explain the relative difference in genetic diversity between the two viruses [138,139]. Also, administration of reverse transcriptase inhibitors was found to not reduce proviral loads, even when administered shortly after infection [140,141].

The most compelling evidence in favor of a clonal expansion of HTLV-1 proviruses is the clonality of T-cells in infected individuals. HTLV-1 integrates randomly into the genome [142,143]. When the HTLV-1 proviral sites are amplified by PCR, it has been consistently found that the T-cells are clonal in both symptomatic and asymptomatic carriers [144,145]. In some HTLV-1 infected individuals, more than one in every 1500 peripheral blood mononuclear cells (PBMCs) were found to be clonal [146].

Since HTLV-1 persistence depends to some extent on the clonal expansion of infected T-cells, it is not surprising that HTLV-1 encodes for gene products that have been found to increase cell proliferation. The oncogenic potential of HTLV-1 is likely a byproduct of the induction of these cellular proliferative pathways. In fact, expression of the HTLV-1 basic leucine zipper (HBZ) and Tax proteins in double transgenic mice was recently shown to be sufficient for the development of lymphoma in the absence of any other viral genes [147]. Since similar pathways influence both cell immortalization and cellular transformation, these two processes are difficult to uncouple. 

### 4.1. Tax

Tax has been established to have many roles in the HTLV-1 proliferative cycle, including essential roles in mediating transcription of HTLV-1 genes as well as the recruitment of the MTOC to sites of cell-cell contact. Tax can also interfere with host cell cycle regulation, apoptotic pathways, and proliferative pathways through a variety of mechanisms. The most commonly studied pathway through which Tax increases cellular proliferation is via the NF-κB/Rel family of proteins.

### 4.2. Tax and Canonical NF-κB Signaling

The NF-κB/Rel proteins are transcription factors that share a Rel homology domain responsible for DNA binding and dimerization. There are five proteins in this family: RelA (p65), RelB, c-Rel, p105 (processed to p50), and p100 (processed to p52). When the NF-κB pathways are turned off, NF-κB dimers are bound to the inhibitor of κB (IκB) proteins. Activation of NF-κB can occur through a canonical pathway in which nuclear localization of NF-κB is induced by inflammatory stimuli such as tumor necrosis factor (TNF) or through a non-canonical pathway reviewed in [148]. 

NF-κB is constitutively active in HTLV-1 infected cells, an abnormality due to the tight regulation of NF-κB by its inhibitor, IκB [149]. IκB kinase (IKK) phosphorylates IκB molecules, leading to the eventual ubiquitination and degradation of IκB and freeing NF-κB dimers to traffic to the nucleus. HTLV-1 Tax increases the activity of IKK by regulating a variety of upstream effectors. Tak1 is a kinase that can phosphorylate and activate IKK. Tax binds directly to Tak1 to form an IKK-Tak1-Tax complex that appears to increase the efficiency of IKK [150]. Tax can also increase the activity of Tak1 by binding to Tak1-binding protein 2 (TAB2); the physiological function of this remains unclear [151]. Tax can also bind directly to TRAF6 and can stimulate its function, leading to the activation of NF-κB through IKK phosphorylation [152]. Finally, Tax can also stimulate MEKK1, which is an upstream regulator of TAK1 [153]. 

In addition to its upstream effects on IKK, Tax also binds directly to an IKK subunit, IKKγ (referred to as NEMO) [154,155]. IKKγ is a regulatory subunit that modulates IKK activity through an unknown mechanism [156]. Protein phosphatase 2A (PP2A) is a positive regulator of IKKγ, and PP2A binding to helix 2 (HLX2) is necessary for proper IKK function [157]. Tax binds IKKγ in a region two heptads downstream of HLX2 termed coiled-coil region 2 (CCR2). When Tax binds this region, it exposes the HLX2 domain for PP2A binding and simultaneously inactivates PP2A, preventing it from dissociating, resulting in IKK being constitutively active [158,159].

Tax also has the ability to form complexes with NF-κB monomers in the cytoplasm. Tax can bind directly to cytoplasmic RelA (p65) and can induce translocation into the nucleus, leading to high transcriptional activity [151,160,161,162]. This particular protein-protein interaction appears to also rely on the CREB-binding protein (CBP or p300), which facilitates the transcriptional activity of RelA.

A role for Tax that has only recently been described is the recognition and inactivation of the ubiquitin-editing enzyme A20. This enzyme is a negative regulator of the canonical NF-κB pathway. It binds with the regulatory protein TAX1BP1 and E3 ligase Itch to form a complex that inactivates essential NF-κB upstream regulators. Tax binds to TAX1BP1, preventing the interaction of TAX1BP1, A20, and Itch [163,164].

### 4.3. Tax and Non-Canonical NF-κB Signaling

Tax is known to facilitate the non-canonical pathway of NF-κB as well as the canonical pathway traditionally induced by MEKK1 and Tak1. This is an important distinction, as the NF-κB dimers are distinct for each pathway and increase expression of different gene products. For example, when the non-canonical pathway is blocked in the presence of Tax, tumorigenesis is significantly delayed in Tax transgenic mice [165]. IKKα phosphorylates p100, which leads to its ubiquitination and cleavage to p52. Tax facilitates this process by recruiting IKKα to p100 and inducing cleavage [166,167].

### 4.4. Tax and Other Cell Proliferative Pathways

In addition to its interaction with NF-κB, HTLV-1 Tax has roles in a variety of other cell proliferation pathways. In particular, Tax interacts directly or indirectly with cyclin-dependent kinases (CDK) [168,169], phosphoinositide 3-kinase (PI3K) [170], transforming growth factor β-1 (TGFβ-1) [171,172], and p53 [173,174,175]. Through a series of complex interactions, Tax ensures that HTLV-1 replication occurs.

### 4.5. Tax Downregulation

Tax plays a key role in the proliferation of T-cells but is known to be a highly immunogenic protein, and Tax-expressing cells are targeted by cytotoxic T-cells [176,177]. That Tax is expressed only at early time points helps to avoid immune surveillance. In patients with ATL, *tax* mRNA transcripts were only found in 34% of all cases [178], indicating that there are downregulation mechanisms in place to prevent HTLV-1-infected cells from being destroyed by the immune system.

Several mechanisms regulate the downregulation of Tax expression. Many *tax* genes become mutated so that non-functional transcripts are produced [178,179,180]. Cells containing these *tax* gene sequences are more likely to replicate due to decreased immune surveillance. Additionally, the *tax* gene is often methylated, leading to gene silencing [178,181]. Finally, the HTLV-1 bZIP factor (HBZ) works to downregulate transcription of many HTLV-1 genes, including Tax [182].

### 4.6. HBZ

The *HBZ* gene is an important HTLV-1 gene that is consistently associated with ATL. *HBZ* mRNA transcripts have been found in virtually all ATL cells, and it was shown that HBZ plays a key role in the proliferation of T-cells [183]. Interestingly, the *HBZ* mRNA appears to play a different role in T-cell proliferation than the HBZ protein [183]. In mouse T-cells, an HBZ start codon mutant that distinguishes between RNA function and protein function was found to increase T-cell proliferation by inhibiting apoptosis and promoting S-phase entry [184]. *HBZ* RNA attenuates apoptosis by promoting the transcription of Survivin, a caspase inhibitor that prevents apoptosis. While HBZ protein promotes S-phase entry, it can also promote apoptosis through its pro-inflammatory effects [184].

HBZ has been reported to directly or indirectly interact with the following proteins that are essential in CREB-dependent cellular proliferation: CREB, CBP, ATF-1, and ATF-3 [185,186,187,188]. Additionally, HBZ interacts with the Jun family of transcription factors, JunB, JunD, and c-Jun [189,190,191]. The combined activities of Tax and HBZ are essential for cellular proliferation. Taken together, the activities of both HBZ and Tax are essential for the transformation of T-cells. 

## 5. Conclusions and Future Directions

As the first human retrovirus discovered in the early 1980s, HTLV-1 has been studied extensively, yet there is still no treatment or vaccine for HTLV-1 infection. Additionally, ATL and HAM/TSP treatments are symptom-based and do not directly treat the viral infection. Continued research on the molecular aspects of HTLV-1 replication will enhance opportunities for the discovery of potential antiretroviral targets that can be exploited for the development of effective therapeutic strategies. For example, a recent candidate peptide (HBZ_157–176_) has been described for use in vaccine development [192]. Such observations help to enhance the likelihood for specific forms of therapeutic intervention for the treatment of HTLV-1 infection. 
